# Executive function development between ages 2 and 6 in children exposed to threatened preterm labor

**DOI:** 10.1007/s00787-026-03115-2

**Published:** 2026-06-29

**Authors:** Anna Miró-Padilla, Belén Almansa, Maite Ferrin, Marta Lizarán, Julia Andreu-Martínez, Adrián Torres-Villasante, Cristina Zapata-de Miguel, Julia Buesa, Alba Moreno-Giménez, Farah Ghosn, Vicente Diago, David Hervás, Ana García-Blanco

**Affiliations:** 1 Neuropsychology and Functional Neuroimaging Group, Department of Basic Psychology, Clinical Psychology and Psychobiology, Universitat Jaume I, Castelló de la Plana, Spain; 2 Mental Health Research Group, Health Research Institute La Fe, Avda. de Fernando Abril Martorell, 106, Valencia, 46026 Spain; 3 Department of Personality, Evaluation and Psychological Treatment, Universitat de València, Valencia, Spain; 4 North London NHS Foundation Trust and ReCognition Health, London, UK; 5 Division of Psychiatry and Clinical Psychology, La Fe University and Polytechnic Hospital, Avenida Fernando Abril Martorell 106, Valencia, 46026 Spain; 6 Centre for Brain and Cognitive Development, Birkbeck College, University of London, London, UK; 7 Division of Obstetrics and Gynecology, La Fe University and Polytechnic Hospital, Valencia, 46026 Spain; 8 Applied Statistics and Operations Research, and Quality, Universitat Politècnica de València, Valencia, Spain

**Keywords:** Threatened preterm labor, Executive function, Longitudinal study, Gestational age, Maternal mental health, Psychosocial risk

## Abstract

Children born after threatened preterm labor (TPL) are at increased risk for executive function (EF) difficulties, even when born at term. However, EF development in this population remains understudied, particularly regarding the role of gestational age and early psychosocial adversity. Prenatal stress (e.g., maternal anxiety at TPL diagnosis and trauma history) and postnatal environmental factors may contribute to variability in EF outcomes. In this prospective cohort study with a 6-year follow-up, EF was assessed at ages 2 and 6 using the Behavior Rating Inventory of Executive Function–Preschool Version (BRIEF-P). The sample included 180 mother–child dyads: 120 children born after TPL (full-term *n* = 27, late-preterm *n* = 56, very-preterm *n* = 37) and 60 full-term controls. Bayesian mixed-effects models were used to examine associations between EF outcomes and gestational age group, time, and relevant prenatal and postnatal factors, including child sex, parental education, maternal trauma history, and maternal anxiety. The models showed a group × time interaction for global EF scores. At age 2, all TPL groups showed higher EF difficulty scores compared with controls, with the largest differences observed in the very-preterm group. Between ages 2 and 6, EF scores decreased in TPL groups relative to controls, indicating a greater reduction in EF difficulties over time, although patterns varied across EF domains. Higher maternal anxiety (prenatal and postnatal), greater maternal trauma exposure, and lower parental education were associated with higher EF difficulty scores, whereas female sex was associated with lower scores. Children born after TPL show early EF vulnerabilities across gestational ages, including those born at term. EF outcomes change across early childhood and are associated with both biological and environmental factors. These findings support the importance of early monitoring of children exposed to prenatal complications and highlight the role of perinatal maternal mental health and family context in early executive function development.

**Supplementary Information:**

The online version contains supplementary material available at https://doi.org/10.1007/s00787-026-03115-2.

## Introduction

Executive functions (EF) comprise a suite of higher-order cognitive processes essential for goal-directed behavior. These include inhibitory control, working memory, cognitive flexibility, planning, and emotional regulation [1, 2]. By integrating these functions, children can effectively regulate attention, manage impulses, and adapt to shifting environmental demands. Such skills are foundational for adaptive functioning across development, particularly within family, social, and educational environments [1, 2]. Critically, EF difficulties—such as poor impulse control, reduced working memory capacity, difficulties shifting between tasks, impaired planning and organization, or dysregulated emotional responses—have been associated with later academic achievement, social competence, and mental health outcomes [1, 3].

Because EF rely heavily on the maturation of prefrontal neural networks, their developmental foundations emerge during fetal life and continue rapidly throughout early childhood [1]. This developmental period is therefore especially sensitive to prenatal adversities, which may disrupt early neurodevelopmental processes, increasing the likelihood of later EF difficulties [1, 3]. In this context, examining EF development in children exposed to early obstetric risk constitutes a key challenge in developmental science. This work examines EF development between ages 2 and 6 in children exposed to threatened preterm labor (TPL), a common obstetric complication associated with increased neurodevelopmental risk, independently of gestational age at birth [4–6]. Focusing on this population allows us to explore early EF development in a high-risk group and to contextualize these findings relative to established research on children born preterm.

Among early biological risk conditions, preterm birth has traditionally been regarded as a major risk factor for EF impairment. Children born very preterm (< 32 weeks of gestation) consistently exhibit greater difficulties in inhibition, working memory, and attentional control compared to full-term peers, and these difficulties may persist into later childhood and adolescence [7–9]. However, EF outcomes among preterm populations are heterogeneous. Increasing evidence indicates that children born moderate-to-late preterm (32–36 weeks) and even early term (37–38 weeks) may also present subtler but clinically meaningful neurocognitive and executive challenges during childhood [10–12]. Moreover, findings from adolescent cohorts suggest that moderate-to-late preterm individuals may continue to exhibit differences in executive and attentional functioning compared to full-term peers, with outcomes typically intermediate between very preterm and full-term groups [13]. Taken together, these findings suggest that gestational age alone does not fully account for the variability observed in EF development, pointing to the potential role of additional prenatal factors.

Beyond prematurity itself, emerging research suggests that neurodevelopmental risk may originate during the prenatal period, particularly in the context of obstetric complications such as TPL [4–6, 14]. TPL is characterized by uterine contractions and cervical changes occurring before 37 weeks of gestation. Importantly, TPL does not always result in preterm birth, with approximately one third of cases ultimately delivering at term [15]. This means that children born at term after a TPL episode may nonetheless have been exposed to prenatal biological and psychosocial stressors capable of influencing early brain development [4]. Longitudinal studies have linked TPL exposure to increased rates of psychomotor delays, emotional dysregulation, attention-deficit/hyperactivity disorder (ADHD) symptoms, and autism-related traits [4–6, 14, 16, 17]. Given the well-established role of EF in self-regulation and in neurodevelopmental conditions such as ADHD and ASD [18, 19], examining EF development may provide insight into mechanisms underlying TPL-related risk.

The mechanisms through which TPL exposure may influence EF development are likely multifactorial. Biologically, TPL has been associated with inflammatory activation, oxidative stress, and alterations in hypothalamic–pituitary–adrenal (HPA) axis functioning, all of which may affect fetal brain maturation [20–24]. In addition, elevated maternal anxiety and psychological distress during pregnancy—particularly at the time of TPL diagnosis—have been associated with alterations in fetal neurodevelopment, potentially through stress-related endocrine pathways [25–27]. Maternal history of trauma and cumulative life stress before conception may further contribute to allostatic load, increasing vulnerability to obstetric complications and influencing early neurodevelopmental outcomes [28–30].

Postnatal environmental factors may also shape EF development following prenatal risk. Parental educational level, socioeconomic context, and maternal mental health during early childhood have been consistently associated with individual differences in EF [7, 26, 31–33]. Rather than acting in isolation, prenatal biological risk and postnatal environmental conditions may both contribute to developmental outcomes. Examining both sets of factors is therefore essential to understanding variation in EF development among children exposed to TPL.

The present study examined developmental change in EF between ages 2 and 6 years in children exposed to TPL, considering gestational age at birth (< 32 weeks; 32 to < 37 weeks; and ≥ 37 weeks) and comparing them with full-term controls without obstetric complications. By assessing EF at two time points during early childhood, we aimed to characterize group differences at age 2 and evaluate whether these differences change by age 6. In addition, we investigated whether prenatal factors (maternal anxiety at TPL diagnosis and maternal trauma history) and postnatal factors (maternal anxiety during early childhood and parental educational level) are associated with EF outcomes across this developmental period. We hypothesized that, at age 2, children exposed to TPL would show greater EF difficulties than full-term controls, with a gradient according to gestational age, such that very preterm TPL children would present the highest levels of difficulty, followed by moderate-to-late preterm TPL, while full-term TPL children would show intermediate levels, potentially comparable to those observed in late-preterm populations [8–10]. We further hypothesized that EF difficulties would decrease between ages 2 and 6 across all TPL groups, reflecting developmental maturation and potential catch-up effects, although residual difficulties would persist, particularly in the very preterm TPL group [7, 34–36]. Finally, we hypothesized that higher levels of prenatal maternal anxiety and trauma exposure, as well as higher postnatal maternal anxiety [25–28], would be associated with higher EF difficulties, whereas higher parental educational attainment would be associated with more favorable EF outcomes across this developmental period [31–33].

## Materials and methods

### Participants

This prospective cohort study followed mother-child pairs from pregnancy through 6 years of age, recruited from the Obstetrics Hospitalization Unit during the mothers’ pregnancies. Pregnant women with TPL were enrolled during their hospitalization, while controls were recruited during routine glucose screening tests between 24 and 28 weeks of gestation. Based on gestational age at birth, children exposed to TPL were classified into three week-range groups: very preterm (< 32 weeks; VP-TPL, *n* = 37), moderate-to-late preterm (32 to < 37 weeks; LP-TPL, *n* = 56), and full-term birth after TPL (≥ 37 weeks; FT-TPL, *n* = 27). The full-term controls consisted of children born full-term without obstetric complications. Mother–child pairs were followed prospectively with assessments during pregnancy, at birth, at 2 years, and at 6 years of age. The study received approval from the institution’s research ethics committee (reference number: 2015/0086). Written informed consent was obtained from both biological parents during pregnancy, before participation in study procedures.

**Fig. 1:**
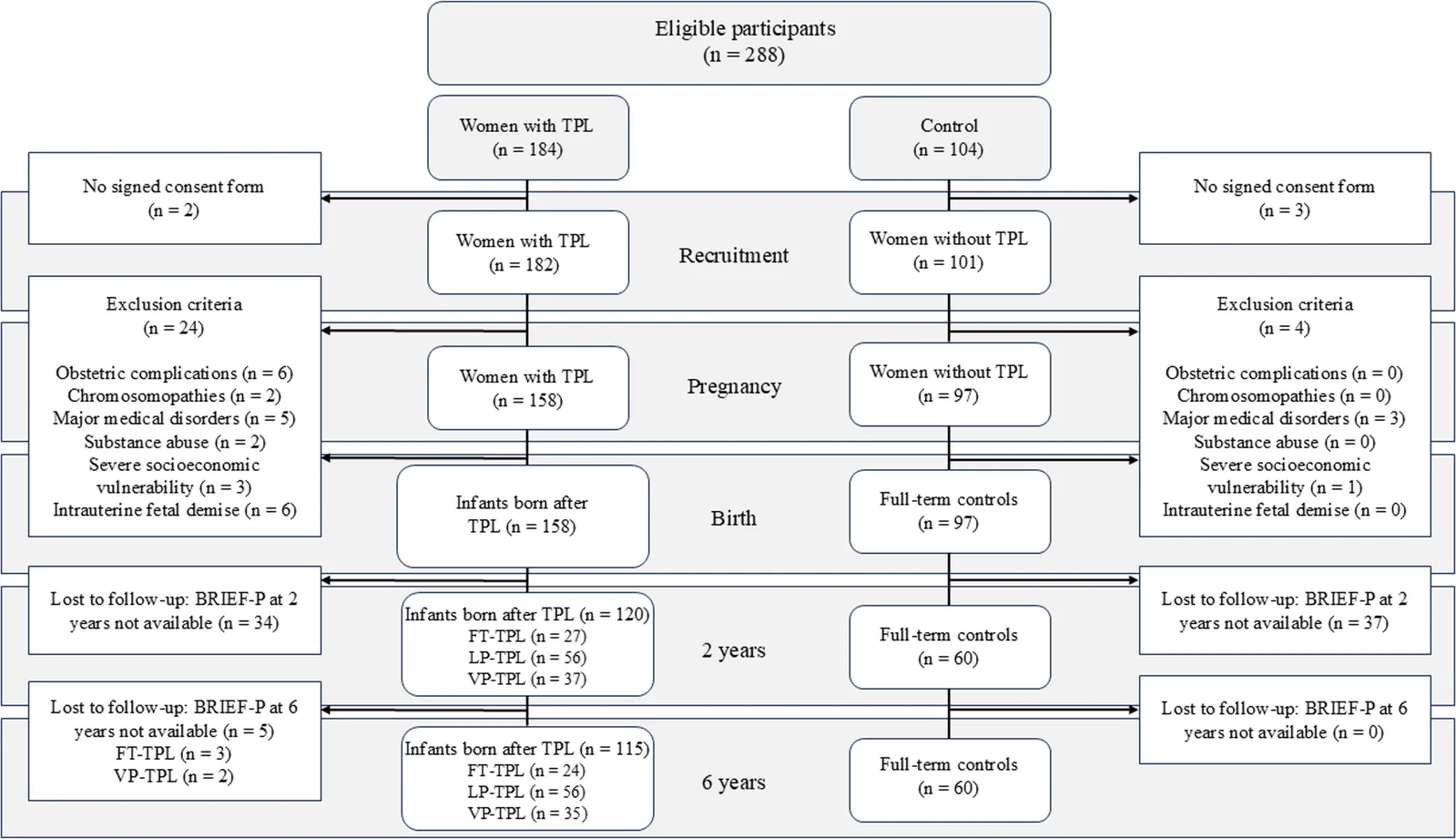
Flowchart of participant recruitment and follow-up TPL = threatened preterm labor; FT-TPL = full-term children born after a TPL; LP-TPL = moderate-to-late preterm children born after a TPL; VP-TPL = very preterm children born after a TPL; BRIEF-P = Behavior Rating Inventory of Executive Function–Preschool Version

Due to the clinical nature of the study, a convenience sampling method was employed. The sample size was determined by the total number of mother-child pairs who met the inclusion criteria and was consecutively recruited over the study period. Recruitment was conducted in a Level III perinatal referral unit specialized in the management of high-risk pregnancies and severe obstetric complications. This approach resulted in a final sample of 180 pairs, providing a clinical cohort of pregnancies complicated by TPL recruited consecutively during the study period (*n* = 120) and a comparable full-term control group (*n* = 60). Although the final sample size was initially determined by the number of eligible participants consecutively recruited, a sample of 180 participants is also statistically adequate for our analyses. A multiple regression including up to 10 predictors (main effects plus interactions) and a medium effect size (Cohen’s f² = 0.15) would require approximately 74 participants. The repeated EF measurements at ages 2 and 6 increase the effective number of observations, providing ample power to detect clinically meaningful group differences and developmental patterns, while allowing robust estimation despite multiple predictors and moderate attrition. Participant recruitment, exclusions, and follow-up across study phases are summarized in Fig. 1.

### Clinical definition of threatened preterm labor

TPL was defined as the presence of regular and painful uterine contractions before 37 weeks of gestation, accompanied by cervical changes, including cervical shortening (< 25 mm), with intact membranes. Women with TPL were recruited between 24 and 32 weeks of gestation during hospitalization for clinical management. This gestational range was selected to ensure homogeneous exposure to magnesium sulfate, administered as standard care for fetal neuroprotection in cases at risk of imminent delivery (cervical dilation ≥ 4 cm with progressive dilation and effacement) before 32 weeks [37], thereby minimizing potential confounding effects.

### Exclusion criteria

Maternal exclusion criteria included severe obstetric complications (e.g., placental abruption, intrauterine growth restriction, preeclampsia, obstructed labor, or signs of infection), major medical conditions requiring chronic treatment, current DSM-5 axis I psychiatric disorders, substance abuse or dependence (including tobacco), and significant language barriers preventing questionnaire completion. Additionally, mothers at risk of severe socioeconomic vulnerability (e.g., lack of stable housing, extreme poverty, or lack of a support network) were excluded to minimize the impact of environmental confounding factors on children’s developmental outcomes [38]. Children with congenital malformations, chromosomal disorders, severe neurological conditions, or major postnatal impairments were also excluded.

### Assessment procedure

The study followed a longitudinal design with data collection organized into four phases: (1) Pregnancy (Recruitment/TPL diagnosis): sociodemographic and obstetric data were collected via semi-structured interviews and medical record reviews. Mothers completed the State-Trait Anxiety Inventory (STAI-S/T), the Beck Depression Inventory (BDI), and the Trauma Questionnaire (TQ). (2) Birth: perinatal and obstetric variables (e.g., birthweight and gestational age) were recorded. (3) Age 2: first assessment of child executive function (EF), in which parents completed the Behavior Rating Inventory of Executive Function—Preschool Version (BRIEF-P). Mothers also completed updated measures of anxiety and depression (STAI-S/T and BDI). (4) Age 6: longitudinal follow-up assessment of child EF using the BRIEF-P, together with updated maternal measures of anxiety and depression (STAI-S/T and BDI). This structured timeline enabled the integration of maternal psychological health, perinatal risk, and long-term developmental outcomes.

### Executive function assessment

EF was assessed using the BRIEF-P, a parent-reported questionnaire comprising 63 items that evaluate executive behaviors in everyday contexts. It generates scores for five EF domains (inhibition, shifting, emotional control, working memory, and planning/organization) as well as a Global Executive Composite (GEC). Higher scores indicate greater executive difficulties. Information regarding participant follow-up and attrition across study phases is summarized in Fig. 1. Missing data were handled within the Bayesian mixed-effects framework, which allows inclusion of participants with incomplete repeated measures.

### Statistical analysis

Numerical variables were summarized using mean (standard deviation) and median (interquartile range), while categorical variables were summarized using frequencies and percentages. Associations between executive function outcomes (BRIEF-P scores) and explanatory variables were assessed using Bayesian mixed-effects models [39], specifically chosen for their robust handling of unbalanced longitudinal data and missing data. These models were used to account for the longitudinal structure of the data, with repeated EF measurements at ages 2 and 6 nested within individuals. All models included a random intercept for participant to account for within-subject dependence. The EF outcomes analyzed were the Global Executive Composite (GEC) and the BRIEF-P subscales (inhibition, shifting, emotional control, planning/organization, and working memory). Predictor variables included group (FT-control, FT-TPL, LP-TPL, VP-TPL), time (2 vs. 6 years), child sex, parental educational level, maternal trauma history (TQ score), maternal anxiety at TPL diagnosis (prenatal STAI-S), and maternal anxiety at follow-up (postnatal STAI-S). Models included fixed effects for group, time, and the group × time interaction to evaluate differences in developmental change between groups. Estimates are reported as posterior means with 95% credible intervals. In all models, the full-term control group at age 2 was used as the reference category. Therefore, group coefficients at T2 represent differences between each TPL group and controls at age 2. The coefficients at T6 correspond to group × time interaction terms and represent the additional change from age 2 to age 6 in each TPL group relative to the change observed in the control group (“Time at 6 years” coefficient). The probability of direction (pd) was used as an index of effect existence, where higher values indicate a higher probability of the existence of a non-null effect [40]. Bayesian mixed-effects models were selected because they allow the inclusion of participants with incomplete repeated measures without requiring listwise deletion of cases with partially missing longitudinal data. Model comparisons were performed using leave-one-out cross-validation. All analyses were conducted in R (version 4.4.2) and the R packages brms (version 2.22.0), bnlearn (version 5.0.1), and bayestestR (version 0.15.0).

## Results

Characteristics of the sample are summarized in Table 1, stratified by group. As expected, gestational age and birthweight differed across groups, reflecting the clinical classification. Despite these differences, birthweight percentiles were broadly comparable, suggesting similar intrauterine growth across groups. No relevant differences were observed in maternal or paternal age. However, the proportion of parents with university education was lower in the TPL groups compared to controls. Notably, pregnancies complicated by TPL showed higher levels of maternal anxiety at recruitment. Differences were also observed in neonatal outcomes, with lower Apgar scores and higher rates of neonatal hospitalization and intensive care unit admission in preterm groups, particularly among children in the VP-TPL group.

**Table 1:** Description of the sample

Variable	Control (*n* = 60) Mean (SD)/*n*(%) Median (1st, 3rd Q.)	FT-TPL (*n* = 27) Mean (SD)/*n*(%) Median (1st, 3rd Q.)	LP-TPL (*n* = 56) Mean (SD)/*n*(%) Median (1st, 3rd Q.)	VP-TPL (*n* = 37) Mean (SD)/*n*(%) Median (1st, 3rd Q.)
Maternal age	32.8 (3.9)	34.4 (4.4)	33 (4.6)	32.5 (4.5)
	33 (31, 36)	34 (32, 37)	33 (29, 36)	33 (30, 36)
Paternal age	36.2 (3.8)	35.4 (4.5)	35.2 (5)	36.7 (6.2)
	36 (34, 39)	36 (34, 38)	35 (31.5, 39.5)	36.5 (32.5, 40.2)
Birthweight (g)	3290.2 (404.1)	3016.4 (478.2)	2083 (430.5)	1201.1 (456.6)
	3275 (3003.7, 3573.7)	3030 (2705, 3287.5)	2050 (1832.5, 2297.5)	1195 (936.2, 1345)
Birthweight percentile for gestational age	47.8 (28.7)	42.8 (30.8)	35.5 (28)	43.8 (32.3)
	46.5 (20.2, 73.7)	39 (15.5, 55.5)	32 (10, 55)	43 (12, 67.7)
Gestational age at birth (week)	39.8 (1.0)	38.4 (1.2)	34.4 (1.4)	28.7 (2.5)
	40 (39.1, 40.4)	38.4 (37.2, 39.2)	34.2 (33.2, 36)	29 (27.2, 31.2)
Recruitment week	37.8 (0.8)	29.3 (3)	30.6 (2.6)	27.4 (2.5)
	38 (38, 38)	30 (28, 31.5)	31.0 (29, 33)	28.0 (25, 29.7)
Apgar score at 1 min	8.9 (1.6)	9.3 (0.67)	8.6 (1.3)	6.8 (2.2)
	9 (9, 10)	9 (9, 10)	9 (9, 9)	7 (6, 9)
Apgar score at 5 min	9.8 (0.4)	9.9 (0.3)	9.8 (0.4)	8.7 (1.3)
	10 (10, 10)	10 (10, 10)	10 (10, 10)	9 (8, 10)
Number of pregnancies	1.8 (0.8)	1.7 (1.1)	1.6 (1.2)	1.5 (1.3)
	2 (1, 2)	1 (1, 2)	1 (1, 2)	1 (1, 1)
Number of deliveries	0.5 (0.5)	0.3 (0.6)	0.4 (0.9)	0.3 (0.9)
	1 (0, 1)	0 (0, 1)	0 (0, 1)	0 (0, 0)
STAI-T score at recruitment	14.9 (7.2)	16.4 (7)	16.7 (8.9)	17.2 (9.6)
	14.5 (9, 19.5)	17 (10.5, 21)	16 (11, 22)	17 (9.5, 20.5)
STAI-S score at recruitment	13.2 (7.3)	21 (10.6)	21.2 (10)	23.7 (11)
	12 (8, 16.2)	21 (13.5, 28.5)	19 (14, 28.5)	21 (15.2, 32)
BDI score at recruitment	2.13 (2.3)	2.48 (3.3)	2.25 (1.9)	3.24 (4.4)
	2 (0.7, 3)	1 (0, 3.5)	2 (1, 3)	1.5 (0, 5)
TQ score at recruitment	1.4 (2.7)	1.1 (1.9)	1.8 (3.2)	4.5 (5.7)
	0 (0, 2)	0 (0, 1)	0 (0, 2.5)	1 (0, 6.5)
Multiple Pregnancy	0 (0)	0.3 (0.4)	0.6 (0.4)	0.5 (0.5)
	0 (0, 0)	0 (0, 1)	1 (0, 1)	1 (0, 1)
Infant sex: Female	29 (48.3%)	15 (55.6%)	24 (42.8%)	17 (45.9%)
Breastfeeding				
Artificial	10 (16.7%)	6 (28.5%)	19 (36.6%)	12 (33.3%)
Maternal	50 (83.3%)	13 (61.9%)	31 (59.6%)	22 (61.1%)
Mixed	0 (0%)	2 (9.5%)	2 (3.8%)	2 (5.5%)
Intensive Care Unit	0 (0%)	0 (0%)	9 (16%)	28 (75.6%)
Neonatal Hospitalization	5 (8.3%)	1 (4.5%)	41 (73.2%)	36 (100%)
In Vitro Fertilization	4 (6.7%)	10 (37%)	14 (25%)	21 (56.7%)
Marital Status				
Heterosexual couple	56 (94.9%)	23 (85.1%)	50 (89.3%)	35 (94.5%)
Homosexual couple	0 (0%)	2 (7.4%)	2 (3.6%)	0 (0%)
Single Mom	0 (0%)	2 (7.4%)	0 (0%)	0 (0%)
Divorced	3 (5%)	0 (0%)	4 (7.1%)	2 (5.4%)
University Education	32 (53.3%)	13 (48.1%)	23 (41%)	15 (40.5%)
Parity: Primiparous	28 (46.7%)	19 (70.4%)	38 (67.8%)	31 (83.7%)
Rescue doses of antenatal corticosteroids	0 (0%)	13 (48.1%)	26 (46.4%)	12 (34.2%)
**BRIEF-P Scales at 2 years**				
Global Executive Composite	87.7 (13.2)	94.8 (12.6)	96.9 (15.4)	102.7 (18.8)
	88 (76, 96.2)	95 (88, 98.5)	95 (86, 107.2)	102 (91, 116)
Emotional Control	13.8 (2.6)	15.2 (3.3)	15.7 (3.8)	15.3 (3.1)
	14 (12, 15)	15 (13, 17)	15 (12.7, 18)	15 (13, 18)
Inhibition	23.3 (4.4)	26.3 (5.4)	25.7 (4.3)	27.3 (6.3)
	22.5 (20, 26)	26 (22, 28)	25 (23, 29)	27 (22, 32)
Working Memory	23.2 (4.6)	24.6 (4)	25.2 (5.1)	29.2 (7)
	23 (19.7, 26)	25 (21.5, 26.5)	24 (21, 28.2)	29 (25, 34)
Shifting	13 (2.5)	14 (2.5)	14.6 (2.9)	14.2 (3.2)
	13 (11, 14.2)	13 (12, 16)	14.5 (12, 17)	14 (11, 15)
Planning and organization	14.2 (2.8)	14.5 (2.1)	15.6 (2.9)	16.6 (3.7)
	14 (12, 16)	15 (13, 16)	15 (14, 16.2)	16 (14, 19)
**BRIEF-P Scales at 6 years**				
Global Executive Composite	82 (13.1)	88.1 (17.6)	82.4 (15.3)	91.1 (21.9)
	80 (71, 90)	84 (75.5, 94.5)	82 (69, 92)	85 (75.7, 104.2)
Emotional Control	12.9 (3.1)	14.7 (3.7)	13.1 (3.1)	12.6 (3)
	12 (11, 14)	14 (11.25, 17.75)	12 (10, 15)	11 (11, 13.5)
Inhibition	21.7 (3.8)	22.9 (5.1)	20.9 (3.7)	23.8 (7.4)
	22 (19, 24)	21.5 (19.2, 24.7)	21 (18, 24)	21.5 (19, 27)
Working Memory	21.3 (4)	22.6 (5)	21.8 (4.8)	26.1 (7.1)
	20 (19, 23)	21 (18.2, 25.7)	20 (18, 24)	24.5 (21, 31)
Shifting	12.67 (2.7)	13 (3.1)	12.6 (2.9)	12.7 (2.9)
	12 (11, 14)	12 (11, 14)	12 (10, 14)	12 (10, 14.2)
Planning and organization	13.3 (2.5)	14.9 (3.9)	14 (3.7)	15.9 (4.5)
	13 (11, 15)	14 (12, 17.7)	13 (11, 16)	15.5 (12.7, 17.2)

*FT-TPL* full-term children born after a threatened preterm labor (TPL), *LP-TPL* moderate-to-late preterm children born after a TPL, *VP-TPL* very preterm children born after a TPL, *STAI-T* State-Anxiety Inventory-Trait Subscale, *STAI-S* State-Anxiety Inventory-State Subscale, *TQ* trauma questionnaire, *BRIEF-P* Behavior Rating Inventory of Executive Function - Preschool Version. Recruitment occurred at the time of the O’Sullivan test in the control group and at the time of threatened preterm labor in the TPL groups

**Fig. 2:**
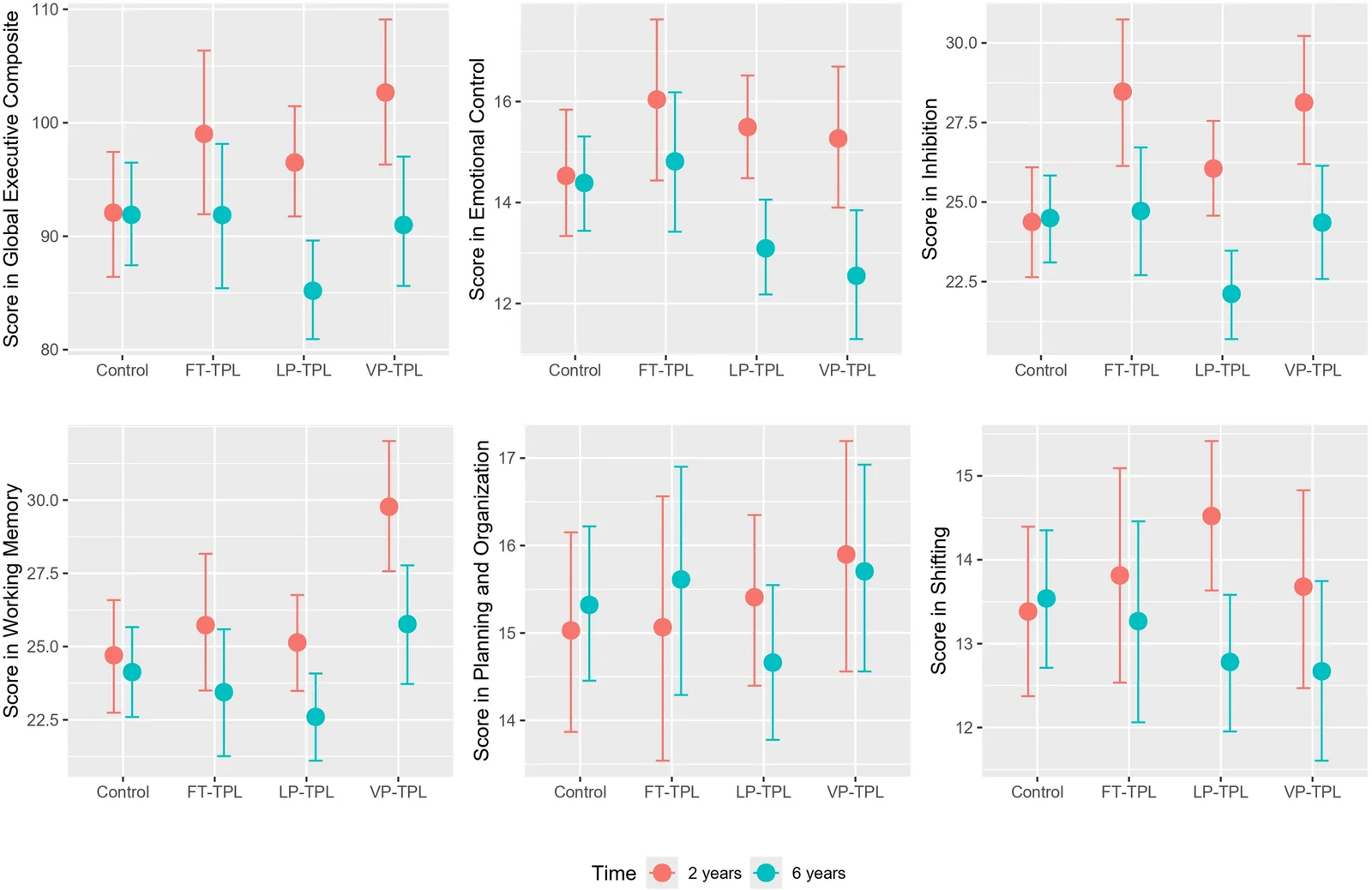
Executive function scores at 2 and 6 years in children born after threatened preterm labor by gestational age groups. FT-TPL = full-term children born after a threatened preterm labor (TPL); LP-TPL = moderate-to-late preterm children born after a TPL; VP-TPL = very preterm children born after a TPL. Points represent model-predicted mean scores for each group and time point. Error bars represent 95% credible intervals derived from the Bayesian mixed-effects models. Scores correspond to raw BRIEF-P scale values (not standardized), where higher scores indicate greater executive function difficulties

Results of the model for GEC are presented in Table 2. The model showed a high probability of direction for the variable group and its interaction with time. All TPL groups showed higher GEC scores than the full-term controls at 2 years (7.17, 4.74, 10.76 points for FT-TPL, LP-TPL, and VP-TPL, respectively, and probabilities of effect of 95.4%, 91.0%, and 99.5%). While the posterior estimate for time in the full-term control group was close to zero, indicating little evidence of change over time, the model indicated that the TPL groups showed a greater reduction in GEC scores between ages 2 and 6 relative to the change observed in the full-term controls. Specifically, the interaction coefficients were − 7.24 points for FT-TPL (pd = 93.0%), − 11.38 points for LP-TPL (pd = 99.8%), and − 11.64 points for VP-TPL (pd = 99.6%), indicating a greater reduction in GEC scores from age 2 to 6 in the TPL groups relative to controls. To facilitate interpretation of this interaction, Fig. 2 illustrates the conditional effects estimated from the adjusted Bayesian mixed-effects model, specifically the interaction between group and time while controlling for the remaining covariates included in the model. The model also showed evidence of association for several covariates. Higher GEC scores were associated with higher prenatal STAI-S scores (increase of 0.29 points in GEC per unit increase, 95% CrI [0.10, 0.47], pd = 99.9%), higher postnatal STAI-S scores (increase of 0.24 points per unit, 95% CrI [0.04, 0.45], pd = 98.8%), and higher trauma questionnaire scores (increase of 0.89 points per unit, 95% CrI [0.33, 1.46], pd = 99.9%). In contrast, lower GEC scores were observed in females compared with males (− 5.39 points, 95% CrI [− 9.50, − 1.29], pd = 99.3%) and in children whose parents had university education (− 7.57 points, 95% CrI [− 11.49, − 3.51], pd > 99.9%).

**Table 2:** Bayesian mixed-effects model for global executive composite

Variables	Estimate	Std.Error	Lower.95.	Upper.95.	Prob. direction
Intercept	82.94	3.87	75.53	90.72	> 99.9%
Female (sex)	−5.39	2.07	−9.51	−1.29	99.3%
Full Term Birth T2	7.17	4.34	−1.67	15.47	95.4%
Moderate-to-late Preterm Birth T2	4.74	3.56	−2.31	11.53	91.0%
Very Preterm Birth T2	10.76	4.19	2.44	18.90	99.5%
Time at 6 years	−0.05	2.88	−5.71	5.63	51.1%
Prenatal STAI-S score	0.29	0.10	0.10	0.47	99.9%
Postnatal STAI-S score	0.24	0.11	0.04	0.45	98.8%
TQ	0.89	0.29	0.33	1.46	99.9%
University education	−7.57	2.07	−11.49	−3.51	> 99.9%
Full Term Birth T6	−7.24	4.84	−16.81	2.21	93.0%
Moderate-to-late Preterm Birth T6	−11.38	3.87	−19.02	−3.61	99.8%
Very Preterm Birth T6	−11.64	4.62	−20.51	−2.54	99.6%

*T2* Time at 2 years, *T6* Time at 6 years, *STAI S* State-Trait Anxiety Inventory-State Subscale, *TQ *Trauma Questionnaire

*FT-TPL* full-term children born after a threatened preterm labor (TPL), *LP-TPL* moderate-to-late preterm children born after a TPL, *VP-TPL* very preterm children born after a TPL. Points represent model-predicted mean scores for each group and time point. Error bars represent 95% credible intervals derived from the Bayesian mixed-effects models. Scores correspond to raw BRIEF-P scale values (not standardized), where higher scores indicate greater executive function difficulties

The emotional control model showed effects in the same direction as those observed for the GEC model, although with weaker overall evidence. At 2 years of age, children in the TPL groups tended to show higher emotional control difficulties than controls, with the largest difference observed in the FT-TPL group (1.50 points, 95% CrI [− 0.34, 3.36], pd = 94%). By 6 years of age, both the LP-TPL and VP-TPL groups showed lower scores relative to controls (LP-TPL: −2.27 points, 95% CrI [− 4.17, − 0.43], pd = 99.3%; VP-TPL: −2.56 points, 95% CrI [− 4.56, − 0.44], pd = 99.2%). As in the GEC model, higher emotional control difficulties were associated with higher prenatal and postnatal maternal anxiety scores and higher maternal trauma scores, whereas children of parents with university education showed lower scores. Full model estimates are provided in Supplementary Table S1.

The inhibition model showed a pattern broadly consistent with that observed for the GEC. At 2 years of age, children in all TPL groups showed higher inhibition difficulties compared with controls, particularly in the FT-TPL and VP-TPL groups. By 6 years of age, all TPL groups showed lower inhibition scores relative to controls, indicating a greater reduction in difficulties over time. Several predictors showed strong evidence of association with inhibition scores, including maternal anxiety (prenatal and postnatal), maternal trauma history, parental educational level, and child sex, with females showing lower scores. Full model estimates are reported in Supplementary Table S2.

The working memory model showed results largely consistent with the previous models. At 2 years of age, only the VP-TPL group showed substantially higher working memory difficulties compared with controls (5.01 points, 95% CrI [2.22, 7.86], pd > 99%). By 6 years of age, a reduction in working memory difficulties relative to controls was observed in the VP-TPL group and, to a lesser extent, in the LP-TPL group. Higher maternal anxiety scores, maternal trauma history, and lower parental educational level were associated with higher working memory scores. Full model estimates are presented in Supplementary Table S3.

The model for planning and organization showed weaker evidence for group differences at 2 years of age, with none of the TPL groups presenting clear differences relative to controls. At 6 years of age, only the LP-TPL group showed a modest reduction in planning and organization difficulties relative to controls. Among the covariates, higher maternal anxiety scores, maternal trauma history, and lower parental educational level were associated with higher scores, whereas females showed slightly lower difficulties. Full results are provided in Supplementary Table S4.

The shifting model provided limited evidence for group differences at 2 years of age, with only the LP-TPL group showing slightly higher difficulties compared with controls. At 6 years of age, reductions in shifting difficulties relative to controls were observed in the LP-TPL and VP-TPL groups. As in the other models, higher maternal anxiety scores and maternal trauma history were associated with higher shifting scores, while parental university education was associated with lower scores. Full model estimates are reported in Supplementary Table S5.

## Discussion

The present study provides novel longitudinal insights into the developmental change in EF in children born after a TPL. As expected, all TPL groups, regardless of gestational age, showed greater EF difficulties at 2 years compared to full-term controls, with the VP-TPL exhibiting the most pronounced impairments. However, unlike previous literature describing persistent EF deficits in preterm populations, our findings revealed a general pattern of improvement between 2 and 6 years across all TPL groups, particularly in the LP group. Still, the evolution of specific EF domains revealed a more heterogeneous picture. Inhibition showed a pattern similar to that of GEC, while working memory deficits remained prominent in VP-TPL children, shifting difficulties were especially marked in LP-TPL children, and emotional control difficulties were more pronounced in the FT-TPL. Inhibition improved consistently across all TPL groups, while planning and organization showed improvement primarily in LP-TPL children, and improvements in working memory, emotional control, and shifting were most evident in LP- and VP-TPL children. Importantly, several prenatal and postnatal environmental factors were associated with higher levels of EF difficulties across early childhood. Specifically, higher maternal anxiety during pregnancy and early childhood, greater maternal trauma exposure, and lower parental educational attainment were consistently associated with higher EF difficulty scores across several domains. Male sex was associated with higher inhibition and planning/organization scores. These findings highlight the dynamic nature of EF development following TPL exposure and underscore the relevance of domain-specific vulnerabilities and the potential modifiability of developmental outcomes through early intervention and environmental support.

At 2 years, all TPL groups exhibited EF difficulties relative to controls, but the severity and domain-specificity of these impairments varied according to gestational age. Our results align with previous research showing that prematurity is a robust risk factor for early EF impairment [8, 9], and that the degree of prematurity is associated with the magnitude of these difficulties [7, 13]. Specifically, VP-TPL children showed the most widespread and severe impairments at 2 years, with deficits observed across nearly all EF domains, especially in working memory and inhibition. This profile is consistent with prior findings on VP populations, which report significant challenges in attention, working memory, and inhibitory control [8, 9]. LP-TPL children also showed EF difficulties, particularly in inhibition and cognitive flexibility, similar to studies that have identified subtler but relevant deficits in these domains among late-preterm children [10, 41]. Interestingly, FT-TPL children, despite being born at term, also demonstrated early vulnerabilities, especially in emotional control and inhibition, echoing recent evidence suggesting that prenatal risk associated with TPL may disrupt early self-regulatory capacities independent of gestational age [4, 16, 23]. These findings support the hypothesis that obstetric complications such as TPL can have EF development consequences even in the absence of preterm birth, underscoring the importance of moving beyond gestational age as the sole marker of early risk.

Our results indicate that children born after a TPL episode show overall improvements in EF between 2 and 6 years, but the pattern of developmental change is shaped by gestational age and EF domain. Contrary to our initial hypothesis, VP-TPL children, despite showing the most pronounced deficits at age 2, exhibited notable progress over time in inhibition, emotional control, working memory, and shifting. However, their difficulties in planning and organization persisted at 6 years, in line with previous longitudinal studies showing that very preterm children often do not fully catch up to their full-term peers in complex EF tasks requiring goal-directed behavior and future-oriented thinking [34–36]. LP-TPL children demonstrated generalized improvement across all domains, suggesting a more favorable EF development. In the FT-TPL group, changes between ages 2 and 6 were more modest and not consistently observed across EF domains, suggesting a less pronounced pattern of improvement over time. These findings suggest that while greater gestational maturity may buffer early vulnerability in EF, it could also reduce the likelihood of receiving specialized neurodevelopmental follow-up, which is typically reserved for preterm populations. In contrast, VP and LP-TPL children may benefit from increased access to early interventions and structured monitoring, as well as enriched postnatal environments that support cognitive development [42–44]. When combined with sensitive caregiving during early developmental stages, characterized by heightened neuroplasticity, these experiences have been associated with better EF development [45]. This may help explain the relative gains observed in the preterm groups. Overall, these results highlight the need to extend targeted support beyond prematurity itself and to include full-term children exposed to prenatal complications such as TPL, who may otherwise remain under the radar despite early regulatory difficulties.

Beyond the role of prematurity, our findings reveal that both prenatal and postnatal factors were associated with differences in EF outcomes across early childhood. Higher levels of maternal anxiety, both during pregnancy and the postnatal period, as well as a maternal history of trauma, were associated with less favorable EF outcomes across early childhood, suggesting a sustained influence on the pace and quality of EF maturation. These results align with longitudinal evidence linking early stress exposures to altered neurodevelopmental patterns, likely via disruptions in fetal programming, HPA axis regulation, and cumulative allostatic load [25, 26, 28, 46]. Nevertheless, these findings contribute novel insights to the existing literature by demonstrating how maternal trauma history and elevated prenatal anxiety not only pose risks to fetal neurodevelopment [27, 30, 47], but also suggest an ongoing influence on EF development across early childhood. While prior studies have established links between these prenatal adversities and later cognitive and behavioral difficulties [48, 49], our longitudinal data highlight their ongoing influence on EF maturation, together with postnatal maternal anxiety, rather than indicating static deficits. Conversely, higher parental educational attainment, particularly at the university level, was associated with more favorable EF growth, supporting prior research on the role of cognitively enriching home environments in promoting executive development [7, 33]. This suggests that postnatal environments can play a critical compensatory role, potentially altering the developmental course initiated by prenatal risk. Furthermore, sex-related differences were observed, with girls showing generally more favorable EF profiles across early childhood [2, 50]. However, smaller differences in shifting, working memory, and emotional control suggest domain-specific variation in the influence of sex, in line with previous longitudinal studies [9]. Overall, these findings highlight the dynamic interplay of biological and environmental factors shaping the development of EF capacities across early childhood.

This study is the first to explore EF development in preschool-aged children born full-term after experiencing a TPL, offering a novel perspective beyond the traditional focus on prematurity. Our longitudinal design enabled the examination of how perinatal complications and maternal anxiety associated with TPL modulate EF development over time, rather than assessing isolated outcomes at a single time point. A notable strength of this work lies in its multidisciplinary framework, integrating expertise from obstetrics, neonatology, psychiatry, psychology, and statistical modeling. Nonetheless, certain limitations must be acknowledged. Our findings are specific to the context of TPL and may not generalize to children exposed to other prenatal adversities or obstetric complications. Furthermore, in an attempt to isolate the specific contribution of TPL-related stress, mothers experiencing severe socioeconomic vulnerability were excluded to reduce the influence of additional environmental prenatal stressors. This approach may have introduced a selection bias toward more socioeconomically advantaged families, thereby limiting the generalizability of the findings to more socially vulnerable populations. Although these criteria reduced sample heterogeneity and sample size, the use of Bayesian mixed-effects modeling allowed us to account for incomplete data and adjust for relevant covariates. Future research would benefit from including parental neurodevelopmental profiles, such as ADHD or ASD symptoms, to better understand intergenerational influences on EF development. Another limitation concerns the exclusive use of parent-reported measures to assess children’s EF. EF outcomes were derived from the BRIEF-P, while some predictors (e.g., maternal anxiety) were also based on maternal self-report. This shared method variance may partially inflate associations between maternal psychological factors and child EF scores. Although the BRIEF-P provides ecologically valid information about children’s everyday executive behavior, future studies would benefit from incorporating multi-informant reports or performance-based EF assessments. In addition, although our longitudinal modeling approach accounts for within-individual continuity across time, we did not examine simple correlations between EF subscales across assessment points. Such analyses could provide complementary information on the stability of EF domains and should be considered in future research.

This longitudinal study provides novel evidence that a TPL event can influence the developmental changes of EF in early childhood, even among children born at term. Our findings show that EF outcomes are not determined solely by gestational age, but emerge from the complex interplay between biological vulnerability and contextual factors associated with EF development, such as prenatal stress, maternal trauma history, postnatal maternal anxiety, and parental educational level. While prematurity remains a major risk factor for EF development, it may also trigger early neurodevelopmental monitoring and access to enriched environments that support partial recovery. In contrast, full-term children exposed to TPL may present subtle but persistent EF vulnerabilities that often go unnoticed and unaddressed in the absence of targeted support. These findings highlight the need to broaden developmental surveillance and early intervention strategies to include all children exposed to prenatal risk, regardless of birth status. Moreover, maternal factors such as anxiety and education showed a significant and ongoing association with EF development, underscoring the importance of early identification and intervention for at-risk mothers and the central role of perinatal mental health services. Future research should investigate whether the observed EF improvements are sustained into later childhood and adolescence, and further clarify the mechanisms underlying developmental adaptation in TPL-exposed children. Understanding these pathways will be key to designing interventions that promote long-term cognitive resilience in children affected by pregnancy complications.

## Supplementary Information

Below is the link to the electronic supplementary material.

Supplementary Material 1 (DOCX 33.8 KB)

## Data Availability

No datasets were generated or analysed during the current study.
