# Supplementary material for: Executive function development between ages 2 and 6 in children exposed to threatened preterm labor
Source: Eur Child Adolesc Psychiatry. 2026 Jun 29;35(11):3497–509. doi: 10.1007/s00787-026-03115-2 (PMC13710616; doi:10.1007/s00787-026-03115-2)
Supplement: Supplementary file 1 [file 787_2026_3115_MOESM1_ESM.docx]

**SUPPLEMENTARY MATERIAL**

Executive Function Development Between Ages 2 and 6 in Children Exposed to Threatened Preterm Labor

Table S1: Bayesian mixed-effects model for **emotional control**.

| **Variables** | **Estimate** | **Std.Error** | **Lower.95.** | **Upper.95.** | **Prob. direction** |
| --- | --- | --- | --- | --- | --- |
| Intercept | 12.87 | 0.83 | 11.23 | 14.45 | 1 |
| Female (sex) | -0.39 | 0.43 | -1.21 | 0.47 | 0.82 |
| Full Term Birth T2 | 1.50 | 0.94 | -0.34 | 3.36 | 0.94 |
| Late Preterm Birth T2 | 0.97 | 0.81 | -0.54 | 2.65 | 0.89 |
| Very Preterm Birth T2 | 0.75 | 0.91 | -1.00 | 2.54 | 0.78 |
| Time at 6 years | -0.12 | 0.68 | -1.40 | 1.28 | 0.57 |
| Prenatal STAI-S score | 0.04 | 0.02 | 0.00 | 0.09 | 0.98* |
| Postnatal STAI-S score | 0.06 | 0.02 | 0.02 | 0.10 | 0.99* |
| TQ | 0.08 | 0.06 | -0.04 | 0.19 | 0.9 |
| University education | -1.15 | 0.41 | -1.96 | -0.33 | 0.99* |
| Full Term Birth T6 | -1.10 | 1.15 | -3.35 | 1.16 | 0.83 |
| Late Preterm Birth T6 | -2.27 | 0.95 | -4.17 | -0.43 | 0.99* |
| Very Preterm Birth T6 | -2.55 | 1.07 | -4.56 | -0.44 | 0.99* |

T2=Time at 2 years; T6=Time at 6 years; STAI S= State-Trait Anxiety Inventory-State Subscale; TQ=Trauma Questionnaire. *pd ≥ 0.975

Table S2: Bayesian mixed-effects model for **inhibition**.

| **Variables** | **Estimate** | **Std.Error** | **Lower.95.** | **Upper.95.** | **Prob. direction** |
| --- | --- | --- | --- | --- | --- |
| Intercept | 22.04 | 1.19 | 19.70 | 24.35 | 1 |
| Female (sex) | -2.31 | 0.61 | -3.49 | -1.14 | 1* |
| Full Term Birth T2 | 4.09 | 1.37 | 1.42 | 6.80 | 0.99* |
| Late Preterm Birth T2 | 1.71 | 1.11 | -0.44 | 3.87 | 0.94 |
| Very Preterm Birth T2 | 3.80 | 1.28 | 1.33 | 6.29 | 0.99* |
| Time at 6 years | 0.13 | 0.98 | -1.83 | 2.06 | 0.552 |
| Prenatal STAI-S score | 0.08 | 0.03 | 0.02 | 0.14 | 0.99* |
| Postnatal STAI-S score | 0.05 | 0.03 | -0.02 | 0.11 | 0.928 |
| TQ | 0.31 | 0.09 | 0.14 | 0.48 | 1* |
| University education | -1.94 | 0.60 | -3.15 | -0.78 | 0.99* |
| Full Term Birth T6 | -3.89 | 1.65 | -6.99 | -0.51 | 0.98* |
| Late Preterm Birth T6 | -4.10 | 1.31 | -6.66 | -1.52 | 0.99* |
| Very Preterm Birth T6 | -3.94 | 1.56 | -7.07 | -0.94 | 0.99* |

T2=Time at 2 years; T6=Time at 6 years; STAI S= State-Trait Anxiety Inventory-State Subscale; TQ=Trauma Questionnaire. *pd ≥ 0.975

Table S3: Bayesian mixed-effects model for **working memory**.

| **Variables** | **Estimate** | **Std.Error** | **Lower.95.** | **Upper.95.** | **Prob. direction** |
| --- | --- | --- | --- | --- | --- |
| Intercept | 22.68 | 1.34 | 20.01 | 25.32 | 1 |
| Female (sex) | -1.41 | 0.73 | -2.86 | 0.00 | 0.974 |
| Full Term Birth T2 | 1.01 | 1.45 | -1.88 | 3.78 | 0.757 |
| Late Preterm Birth T2 | 0.41 | 1.18 | -1.86 | 2.72 | 0.63 |
| Very Preterm Birth T2 | 5.01 | 1.40 | 2.22 | 7.86 | 1* |
| Time at 6 years | -0.58 | 0.93 | -2.35 | 1.23 | 0.739 |
| Prenatal STAI-S score | 0.07 | 0.03 | 0.01 | 0.13 | 0.98* |
| Postnatal STAI-S score | 0.06 | 0.04 | -0.01 | 0.13 | 0.952 |
| TQ | 0.21 | 0.10 | 0.01 | 0.41 | 0.97* |
| University education | -2.21 | 0.71 | -3.57 | -0.83 | 0.99* |
| Full Term Birth T6 | -1.71 | 1.52 | -4.63 | 1.35 | 0.879 |
| Late Preterm Birth T6 | -1.93 | 1.24 | -4.41 | 0.47 | 0.939 |
| Very Preterm Birth T6 | -3.44 | 1.42 | -6.22 | -0.61 | 0.99* |

T2=Time at 2 years; T6=Time at 6 years; STAI S= State-Trait Anxiety Inventory-State Subscale; TQ=Trauma Questionnaire. *pd ≥ 0.975

Table S4: Bayesian mixed-effects model for **planning and organization**.

| **Variables** | **Estimate** | **Std.Error** | **Lower.95.** | **Upper.95.** | **Prob. direction** |
| --- | --- | --- | --- | --- | --- |
| Intercept | 12.98 | 0.79 | 11.41 | 14.52 | 1 |
| Female (sex) | -0.94 | 0.42 | -1.78 | -0.13 | 0.98* |
| Full Term Birth T2 | 0.06 | 0.89 | -1.68 | 1.84 | 0.523 |
| Late Preterm Birth T2 | 0.42 | 0.73 | -0.96 | 1.85 | 0.713 |
| Very Preterm Birth T2 | 0.90 | 0.88 | -0.77 | 2.66 | 0.85 |
| Time at 6 years | 0.34 | 0.60 | -0.84 | 1.52 | 0.712 |
| Prenatal STAI-S score | 0.06 | 0.02 | 0.02 | 0.10 | 0.99* |
| Postnatal STAI-S score | 0.05 | 0.02 | 0.01 | 0.09 | 0.99* |
| TQ | 0.15 | 0.06 | 0.03 | 0.27 | 0.99* |
| University education | -1.22 | 0.41 | -2.06 | -0.44 | 0.99* |
| Full Term Birth T6 | 0.20 | 0.99 | -1.74 | 2.20 | 0.589 |
| Late Preterm Birth T6 | -1.10 | 0.79 | -2.69 | 0.44 | 0.92 |
| Very Preterm Birth T6 | -0.51 | 0.95 | -2.36 | 1.36 | 0.697 |

T2=Time at 2 years; T6=Time at 6 years; STAI S= State-Trait Anxiety Inventory-State Subscale; TQ=Trauma Questionnaire. *pd ≥ 0.975

Table S5: Bayesian mixed-effects model for **shifting**.

| **Variables** | **Estimate** | **Std.Error** | **Lower.95.** | **Upper.95.** | **Prob. direction** |
| --- | --- | --- | --- | --- | --- |
| Intercept | 12.35 | 0.70 | 10.95 | 13.71 | 1 |
| Female (sex) | -0.45 | 0.39 | -1.20 | 0.34 | 0.87 |
| Full Term Birth T2 | 0.48 | 0.80 | -1.07 | 2.07 | 0.71 |
| Late Preterm Birth T2 | 1.17 | 0.64 | -0.08 | 2.44 | 0.96 |
| Very Preterm Birth T2 | 0.27 | 0.75 | -1.17 | 1.75 | 0.63 |
| Time at 6 years | 0.18 | 0.48 | -0.78 | 1.12 | 0.64 |
| Prenatal STAI-S score | 0.03 | 0.02 | 0.00 | 0.07 | 0.98* |
| Postnatal STAI-S score | 0.03 | 0.02 | -0.01 | 0.07 | 0.91 |
| TQ | 0.15 | 0.05 | 0.04 | 0.25 | 0.99* |
| University education | -1.07 | 0.38 | -1.80 | -0.30 | 0.99* |
| Full Term Birth T6 | -0.72 | 0.83 | -2.28 | 0.90 | 0.81 |
| Late Preterm Birth T6 | -1.92 | 0.64 | -3.18 | -0.66 | 0.99* |
| Very Preterm Birth T6 | -1.15 | 0.76 | -2.64 | 0.39 | 0.93 |

T2=Time at 2 years; T6=Time at 6 years; STAI S= State-Trait Anxiety Inventory-State Subscale; TQ=Trauma Questionnaire. *pd ≥ 0.975
